# Patient-reported outcomes after idecabtagene vicleucel vs. ciltacabtagene autoleucel CAR-T for multiple myeloma

**DOI:** 10.1038/s41409-026-02899-w

**Published:** 2026-05-11

**Authors:** Laura B. Oswald, Xiaoyin Li, Lisa M. Gudenkauf, Gabe De Avila, David Scheiber-Camoretti, Brent J. Small, Brian D. Gonzalez, Aasha I. Hoogland, Oanh Nguyen, Yvelise Rodriguez, Sylvia L. Crowder, Nathan H. Parker, Tiffany L. Carson, Christine E. Vinci, Rachid C. Baz, Kenneth H. Shain, Brandon Blue, Ariel Grajales-Cruz, Kristy Matte, Brandon Kale, David Kaldas, Fabiana Perna, Melissa Alsina, Ciara L. Freeman, Omar Castaneda Puglianini, Taiga Nishihori, Hien Liu, Frederick L. Locke, Heather S. L. Jim, Lauren C. Peres, Doris K. Hansen

**Affiliations:** 1 Department of Health Outcomes and Behavior, Moffitt Cancer Center, Tampa, FL USA; 2 Department of Psychiatry, Brigham and Women’s Hospital, Boston, MA USA; 3 Department of Blood and Marrow Transplant and Cellular Immunotherapy, Moffitt Cancer Center, Tampa, FL USA; 4 School of Nursing, University of North Carolina at Chapel Hill, Chapel Hill, NC USA; 5 Department of Malignant Hematology, Moffitt Cancer Center, Tampa, FL USA; 6 Department of Cancer Epidemiology, Moffitt Cancer Center, Tampa, FL USA

**Keywords:** Quality of life, Cancer immunotherapy

## Abstract

This was the first study to assess patient-reported outcome (PRO) trajectories among patients with relapsed/refractory multiple myeloma (RRMM) receiving standard of care chimeric antigen receptor T-cell therapy (CAR-T) and to compare PRO trajectories by treatment group, idecabtagene vicleucel (ide-cel) vs. ciltacabtagene autoleucel (cilta-cel). Participants completed health-related quality of life (HRQOL) and symptom surveys at enrollment/baseline (pre-lymphodepletion), infusion day(D)0, D7, D14, D21, D30, D60, and D90. Piecewise growth curve models assessed PRO trajectories pre-D7 and post-D7. Among 99 participants, ide-cel recipients (*n* = 49) were older than cilta-cel recipients (*n* = 50) (median 73 vs. 64 years, *p* < 0.001). Many PROs worsened pre-D7 and improved post-D7, including overall HRQOL, physical well-being, functional well-being, fatigue, physical function, and social function (*p*-values < 0.05). Several PROs were stable pre-D7 and improved post-D7, including anxiety, sleep disturbance, pain interference, and pain intensity (*p*-values < 0.05). There were differences between groups in the trajectories of social well-being pre-D7 (*p* = 0.01) and cognitive function post-D7 (*p* = 0.001). Patients with RRMM receiving standard of care CAR-T had similar PRO trajectories regardless of CAR-T type, with most PROs initially worsening or stable before significantly improving post-treatment. Future studies should investigate potential differences by treatment for social well-being and cognitive function as well as PRO trajectories beyond D90 post-treatment.

## Introduction

Chimeric antigen receptor T-cell therapy (CAR-T) targeting B-cell maturation antigen (BCMA) is a groundbreaking cellular immunotherapy for patients with relapsed or refractory multiple myeloma (RRMM). Before CAR-T, prior RRMM therapies had an overall response rate of ~32% [1]. In the phase II KarMMa trial, idecabtagene vicleucel (ide-cel) demonstrated a 73% overall response rate (33% complete response or better) [2], and in the phase Ib/II CARTITUDE-1 trial, ciltacabtagene autoleucel (cilta-cel) demonstrated a 98% overall response rate (83% complete response or better) [3, 4]. In long-term follow-up of CARTITUDE-1, one-third of treated patients remained alive and progression-free without maintenance treatment for five years or longer [5]. Accordingly, ide-cel and cilta-cel were FDA-approved as fifth-line therapies for RRMM in March 2021 [6] and February 2022 [7], respectively, and have since been approved as earlier lines of therapy [8, 9].

CAR-T trials also showed benefits for patient-reported outcomes (PROs). PROs are health information reported directly by patients without interpretation by another person [10] and include health-related quality of life (HRQOL) (e.g., overall well-being) and symptom burden. In the KarMMa and CARTITUDE-1 trials, ide-cel and cilta-cel were associated with significant improvements in key PRO domains (e.g., overall HRQOL, fatigue, pain, physical function) as early as one to two months post-infusion, and many improvements were sustained for over one year [11, 12]. Treatment regimens for RRMM are historically less likely to improve HRQOL than first-line therapies [13], making these findings particularly noteworthy. Recently, the phase III KarMMa-3 and CARTITUDE-4 trials showed that ide-cel and cilta-cel recipients had larger PRO improvements than patients treated with standard regimens [14, 15]. Thus, the integration of CAR-T into standard of care has the potential to improve patients’ quantity and quality of life.

Given the recency of these treatment advances, longitudinal PRO studies in the real-world are limited. Real-world studies are critical complements to clinical trials [16, 17] because trial participants tend to be younger and have better overall health than patients in standard of care [18], limiting the generalizability of trial findings. Our team previously published the only longitudinal PRO study of RRMM CAR-T recipients in standard of care, which only included patients receiving ide-cel [19]. Moreover, no studies have compared PROs between CAR-T treatment groups. In recent real-world reports, cilta-cel is associated with better treatment outcomes (i.e., treatment responses, progression-free and overall survival) but higher incidence of certain toxicities (e.g., delayed neurotoxicity) [20, 21]. A study comparing PROs between CAR-T treatments would provide complementary information relevant to patients and clinicians for treatment decision-making and for setting expectations for potential HRQOL changes post-treatment. To address these limitations and expand our prior work, this study assessed trajectories of PROs among RRMM patients receiving ide-cel and cilta-cel in standard of care and compared PRO trajectories between groups.

## Methods

### Ethics approval and consent to participate

All methods were performed in accordance with the Declaration of Helsinki. The protocol was reviewed by Advarra Institutional Review Board and deemed exempt/low risk (Pro00065891). Informed consent was obtained from all participants.

### Participants and procedures

Patients were recruited from Moffitt Cancer Center, an NCI-designated comprehensive cancer center in Tampa, FL, for a prospective observational study. Participants met the following criteria: 1) ≥18 years old, 2) scheduled to receive standard of care ide-cel or cilta-cel for RRMM based on their treating physician’s discretion, 3) able to speak and read English or Spanish, 4) without documented or observable psychiatric or neurologic diagnoses that could interfere with participation (e.g., dementia), and 5) able to provide informed consent. Between September 2022 and June 2024, research coordinators identified potentially eligible patients with assistance from providers in Moffitt’s Immune Cell Therapy program. Coordinators screened patients’ electronic medical records (EMRs) for eligibility and approached patients to introduce the study, confirm eligibility, answer questions, and solicit informed consent. Participants completed PRO assessments electronically via REDCap [22], a HIPAA-compliant and internet-based data capture tool, at baseline (before lymphodepleting chemotherapy), CAR-T infusion day (D)0, D7, D14, D21, D30, D60, and D90. This timeline was informed by routine CAR-T clinical care, expert recommendations for monitoring PROs post-CAR-T, and our work showing feasibility [19, 23, 24]. Study materials were available in English and Spanish (individual preference). Participants were compensated $10 per assessment.

### Measures

#### Demographic and clinical characteristics

At baseline, participants reported their demographics and completed the patient-reported Charlson Comorbidity Index [25, 26]. EMR reviews were conducted for baseline clinical characteristics (e.g., treatment history, Eastern Cooperative Oncology Group (ECOG) performance status), treatment information (e.g., CAR-T regimen), and safety and clinical outcomes through D90 (e.g., physician-reported cytokine release syndrome (CRS) and immune effector cell-associated neurotoxicity syndrome (ICANS) assessed per American Society for Transplantation and Cellular Therapy criteria) [27].

#### PROs

The 27-item Functional Assessment of Cancer Therapy-General (FACT-G) is a validated measure of HRQOL over the past week with four subscales: physical, social, emotional, and functional well-being [28]. Participants responded to items on a Likert-type scale from 0 to 4. Higher scores reflect better HRQOL. Mean scores for overall HRQOL ≤70, physical well-being ≤18, social well-being ≤19, emotional well-being ≤15, and functional well-being ≤14 indicate clinically low HRQOL [29].

The 31-item NIH-developed PRO Measurement Information System (PROMIS)-29 + 2 Profile v2.1 assesses fatigue, depression, anxiety, sleep disturbance, pain interference (with daily life), physical function, social function (4 items each), cognitive function (2 items), and pain intensity (1 item) over the past week [30, 31]. Participants rated their pain intensity from 0 to 10, with higher scores reflecting worse pain. For the remaining scales, participants responded to items on Likert-type scales from 1 to 5 and standardized T-scores were calculated, with normative mean = 50 and standard deviation = 10. Higher scores are worse for fatigue, depression, anxiety, sleep disturbance, pain interference, (56–60 mild, 61–70 moderate, >70 severe) [32], and pain intensity (1–2 mild, 3–5 moderate, ≥6 severe) [33]. Lower scores are worse for physical, social, and cognitive function (<30 severe, 30–49 moderate, 40–44 mild) [32].

### Statistical analysis

Chi-square tests, independent samples t-tests, and Wilcoxon rank sum tests (for non-normally distributed variables) were used to examine differences in participant characteristics and PROs by treatment at baseline. Longitudinal changes in PROs by treatment were evaluated using piecewise growth curve models to account for non-linear trajectories. Time was segmented into two phases: baseline through D7 (pre-D7; acute CAR-T treatment period) and D7 through D90 (post-D7; post-treatment recovery period). D7 was selected to bisect the models because mean PRO scores were worst (e.g., highest for symptoms) at D7 for most PROs, and this represents the transition between short- and longer-term treatment impact. Maximum likelihood estimation was used to incorporate all available PRO data. Participants with missing covariate data were excluded from the piecewise growth curve models. Analyses controlled for high priority/risk demographic and clinical variables identified a priori, including age (years), race and ethnicity (non-Hispanic White, non-Hispanic Black, Hispanic), marital status (married, not married), ECOG performance status (0–1, ≥2), high-risk cytogenetics (any, none), extramedullary disease (yes, no), high bone marrow burden (yes, no), and prior anti-BCMA therapy (any, none). Finally, paired-samples *t*-tests were used to compare average PRO scores at baseline and D90 across treatment groups. Analyses were conducted in R (*lme4* package) [34–36], two-sided, and significant if *p* < 0.05.

## Results

### Participant characteristics

Supplemental Fig. 1 shows participants’ flow through the study. Of 161 eligible patients approached, 99 enrolled and provided at least baseline data for analysis (61% recruitment rate). The most common reasons for non-participation were not being interested in research and feeling overwhelmed while preparing for CAR-T. As shown in Supplemental Table 1, eligible patients who declined participation were more likely to have an ECOG performance status ≥2 (23% vs. 9%, *p* = 0.01) and any high-risk cytogenetic abnormality (60% vs. 37%, *p* = 0.02), defined as the presence of del(17p), t(4;14), and/or t(14;16) any time before CAR-T infusion. There were no other differences in baseline demographic or clinical characteristics between eligible patients who enrolled vs. declined. The sample included 49 patients receiving ide-cel and 50 receiving cilta-cel. Three participants passed away, two formally withdrew, and retention was high (*n* = 89, 93% of evaluable participants; *n* = 44 ide-cel; *n* = 45 cilta-cel).

Supplementary Table 2 shows participants’ baseline demographic and clinical characteristics overall and by treatment. Ide-cel recipients were older than cilta-cel recipients (median 73 vs. 64 years, *p* < 0.001). There were no other differences in baseline demographic or clinical characteristics between groups. Overall, approximately two-thirds of participants were male (61%) and identified as non-Hispanic White (68%), and most were married (80%). Participants had median 5 prior therapies before CAR-T (range 3–10). A minority of participants had high bone marrow burden (24%; defined as ≥50% CD138-positive plasma cells in pre-treatment bone marrow core biopsy), extramedullary disease (15%), ECOG performance status ≥2 (9%), or received prior anti-BCMA therapy (7%). Approximately one-third had any high-risk cytogenetic abnormality (37%), most commonly del(17p) (23%), and three-quarters had received bridging therapy (76%).

Safety and clinical outcomes through D90 are shown in Supplementary Table 3. Ide-cel recipients (vs. cilta-cel) had longer hospitalizations (median 9 vs. 5 days, *p* < 0.001) and lower prevalence of non-ICANS neurotoxicity (0% vs. 14%, *p* = 0.012). There were no other differences in safety or clinical outcomes between groups. Overall, 19% of participants developed grade ≥2 CRS, 11% developed grade ≥2 ICANS, and 88% had a partial response or better by D90.

### Baseline PROs

Unadjusted mean PRO scores by treatment at each timepoint are shown in Supplementary Tables 4, 5. At baseline, average physical function scores indicated mild dysfunction for both groups (ide-cel M = 42.04, cilta-cel M = 44.11). Average pain interference scores were in the mild range for ide-cel (M = 56.29) and within the normal range for cilta-cel (M = 53.93). All other baseline PROs were within normal ranges, and there were no group differences.

### PRO trajectories

**Fig. 1:**
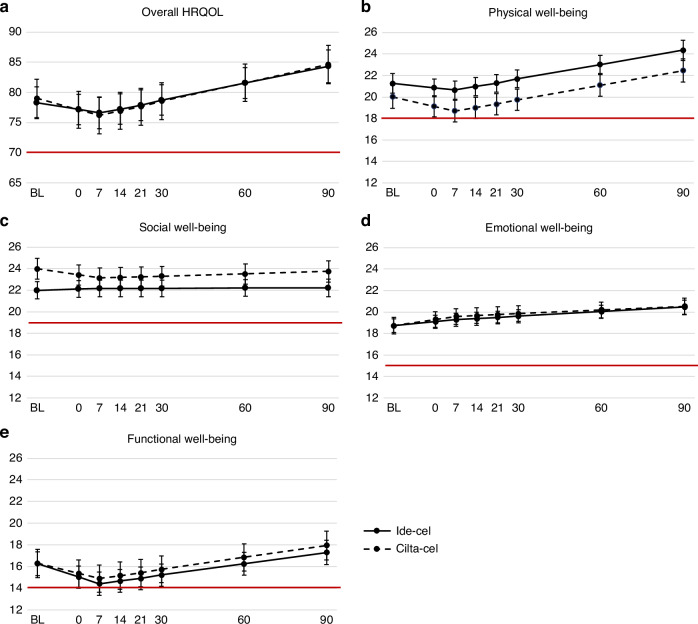
Estimated PRO trajectories by CAR-T treatment group for FACT-G. **a** Overall HRQOL, **b** physical well-being, **c** social well-being, **d** emotional well-being, and **e** functional well-being. Ide-cel is depicted with a solid black line and cilta-cel is depicted with a dashed black line. Higher scores are better. Thresholds for indicating clinically low HRQOL (i.e., below the threshold) are depicted with a red line: overall HRQOL ≤ 70, physical well-being ≤18, social well-being ≤19, emotional well-being ≤15, and functional well-being ≤14. Error bars represent 95% confidence intervals. Estimates for the figures were obtained using a reference dataset where continuous covariates (i.e., age) were set to their mean values and categorical covariates were assigned to the most common category (i.e., non-Hispanic White, married, ECOG performance status <2, no high-risk cytogenetics, no extramedullary disease, no high marrow burden, and no prior anti-BCMA therapy).

**Fig. 2:**
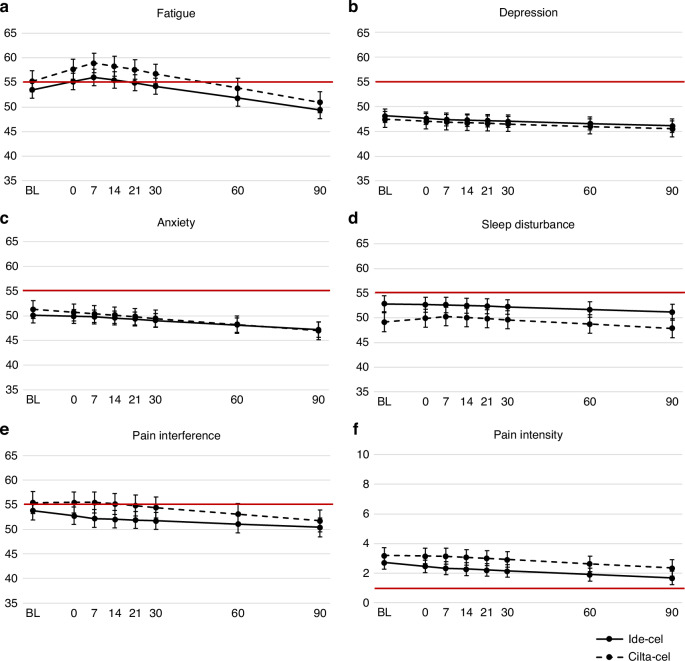
Estimated PRO trajectories by CAR-T treatment group for PROMIS. **a** Fatigue, **b** depression, **c** anxiety, **d** sleep disturbance, **e** pain interference, and **f** pain intensity. Ide-cel is depicted with a solid black line and cilta-cel is depicted with a dashed black line. Higher scores are worse for fatigue, depression, anxiety, sleep disturbance, pain interference (55–59 mild, 60–69 moderate, ≥70 severe), and pain intensity (1–2 mild, 3–5 moderate, ≥6 severe). Thresholds for indicating clinically meaningful scores (i.e., above the threshold) are depicted with a red line. Error bars represent 95% confidence intervals. Estimates for the figures were obtained using a reference dataset where continuous covariates (i.e., age) were set to their mean values and categorical covariates were assigned to the most common category (i.e., non-Hispanic White, married, ECOG performance status <2, no high-risk cytogenetics, no extramedullary disease, no high marrow burden, and no prior anti-BCMA therapy).

**Fig. 3:**
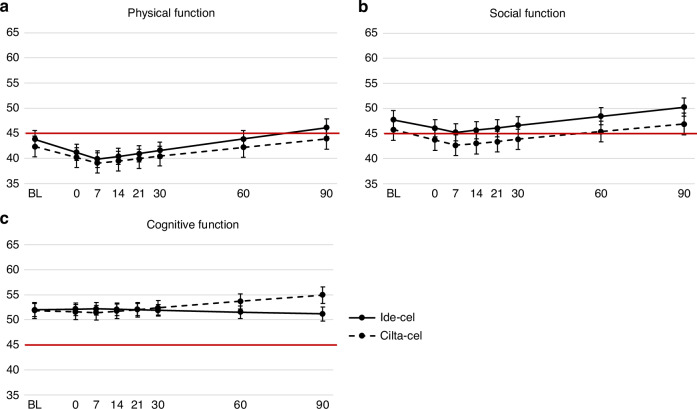
Estimated PRO trajectories by CAR-T treatment group for PROMIS. **a** Physical function, **b** social function, and **c** cognitive function. Ide-cel is depicted with a solid black line and cilta-cel is depicted with a dashed black line. Lower scores are worse (≤30 severe, 31–40 moderate, 41–45 mild dysfunction). Thresholds for indicating clinically meaningful scores (i.e., below the threshold) are depicted with a red line. Error bars represent 95% confidence intervals. Estimates for the figures were obtained using a reference dataset where continuous covariates (i.e., age) were set to their mean values and categorical covariates were assigned to the most common category (i.e., non-Hispanic White, married, ECOG performance status <2, no high-risk cytogenetics, no extramedullary disease, no high marrow burden, and no prior anti-BCMA therapy).

Tables 1–3 show the results of piecewise growth curve models examining PRO changes by treatment. Figures 1–3 show the estimated PRO trajectories by treatment obtained using least-squares means, with continuous covariates (i.e., age) set to their mean values and categorical covariates assigned to the most common category (i.e., non-Hispanic White, married, ECOG < 2, no high-risk cytogenetics, extramedullary disease, high marrow burden, or prior anti-BCMA therapy).

**Table 1:** Results of piecewise growth curve models examining changes in FACT-G total and subscale scores by treatment group over time.

	Overall HRQOL	Physical well-being	Social well-being	Emotional well-being	Functional well-being
(Intercept)	61.92 (39.85, 83.91)***	20.4 (13.44, 27.34)***	13.71 (6.96, 20.44)***	17.23 (11.98, 22.43)***	10.42 (1.29, 19.52)*
Age	0.22 (−0.11, 0.55)	−0.04 (−0.14, 0.07)	0.14 (0.04, 0.24)*	0.04 (−0.04, 0.12)	0.08 (−0.06, 0.21)
Non-Hispanic Black	−0.22 (−7.84, 7.45)	−2.05 (−4.46, 0.38)	0.30 (−2.03, 2.65)	1.37 (−0.44, 3.19)	0.30 (−2.86, 3.47)
Hispanic	3.66 (−3.46, 10.78)	0.87 (−1.37, 3.10)	0.93 (−1.25, 3.11)	0.57 (−1.11, 2.26)	1.27 (−1.67, 4.22)
Married	1.11 (−5.28, 7.48)	1.38 (−0.64, 3.40)	0.28 (−1.67, 2.23)	−0.56 (−2.08, 0.95)	0.02 (−2.63, 2.65)
Performance status ≥2	−9.82 (−19.14, −0.51)	−1.34 (−4.29, 1.61)	−1.35 (−4.20, 1.50)	−1.96 (−4.18, 0.24)	−5.19 (−9.04, −1.34)*
High-risk cytogenetics	−1.35 (−6.88, 4.18)	−0.38 (−2.13, 1.36)	0.02 (−1.68, 1.71)	−0.24 (−1.55, 1.08)	−0.78 (−3.07, 1.51)
Extramedullary disease	5.44 (−1.91, 12.82)	1.61 (−0.70, 3.95)	1.89 (−0.36, 4.14)	0.07 (−1.68, 1.82)	1.95 (−1.09, 5.00)
High marrow burden	4.18 (−2.19, 10.55)	1.66 (−0.35, 3.66)	0.21 (−1.74, 2.16)	0.89 (−0.62, 2.40)	1.40 (−1.24, 4.04)
Prior anti-BCMA therapy	−1.94 (−12.01, 8.12)	−0.74 (−3.91, 2.43)	0.03 (−3.06, 3.10)	−1.28 (−3.67, 1.10)	0.09 (−4.07, 4.25)
Pre-D7	−0.13 (−0.24, −0.03)*	−0.06 (−0.11, −0.01)*	−0.04 (−0.07, −0.01)*	0.04 (0.01, 0.07)**	−0.07 (−0.11, −0.02)**
Post-D7	0.10 (0.07, 0.13)***	0.05 (0.03, 0.06)***	0.01 (<0.01, 0.02)	**0.01 (**<0.01, 0.02)*	0.04 (0.02, 0.05)***
Treatment	0.66 (−5.47, 6.75)	1.86 (−0.12, 3.81)	−1.34 (−3.21, 0.52)	−0.06 (−1.52, 1.39)	0.08 (−2.45, 2.60)
Pre-D7 × Treatment	0.04 (−0.12, 0.20)	0.03 (−0.04, 0.11)	**0.05 (**<0.01, 0.09)*	−0.01 (−0.06, 0.03)	−0.03 (−0.09, 0.04)
Post-D7 × Treatment	<0.01 (−0.05, 0.05)	<0.01 (−0.02, 0.02)	<0.01 (−0.02, 0.01)	<0.01 (−0.01, 0.02)	<0.01 (−0.02, 0.02)

Reference groups for categorical variables are as follows: non-Hispanic White race and ethnicity, not married, performance status <2, no high-risk cytogenetics, no extramedullary disease, no high bone marrow burden, and no prior anti-BCMA therapy. Reference group for treatment was cilta-cel. *n* = 8 participants were excluded from the models due to missing covariate data (*n* = 6 missing high bone marrow burden; *n* = 5 missing high-risk cytogenetics; *n* = 1 missing extramedullary disease, not mutually exclusive) or other race/ethnicity group (*n* = 1).

Values reported are betas with 95% confidence intervals.

Significant values are indicated with bold text; *p < 0.05, **p < 0.01, ***p < 0.001.

**Table 2:** Results of piecewise growth curve models examining changes in PROMIS fatigue, depression, anxiety, sleep disturbance, pain interference, and pain intensity scores by treatment group over time.

	Fatigue	Depression	Anxiety	Sleep disturbance	Pain interference	Pain intensity
(Intercept)	51.67 (37.48, 65.93)***	47.16 (36.62, 57.79)***	52.44 (40.57, 64.39)***	62.93 (49.74, 76.21)***	58.86 (44.01, 73.74)***	3.79 (0.08, 7.51)
Age	0.12 (−0.09, 0.34)	−0.01 (−0.16, 0.15)	0.01 (−0.17, 0.18)	−0.15 (−0.35, 0.04)	−0.03 (−0.26, 0.19)	<0.01 (−0.06, 0.05)
Non-Hispanic Black	−0.49 (−5.51, 4.46)	−2.34 (−6.08, 1.34)	−2.49 (−6.70, 1.67)	−2.76 (−7.41, 1.85)	2.13 (−3.11, 7.31)	0.98 (−0.33, 2.27)
Hispanic	−3.53 (−8.12, 1.05)	−1.70 (−5.11, 1.71)	−2.96 (−6.79, 0.88)	−2.27 (−6.53, 2.00)	−2.09 (−6.86, 2.71)	−0.23 (−1.43, 0.97)
Married	−2.51 (−6.66, 1.65)	−0.04 (−3.12, 3.05)	−2.57 (−6.03, 0.91)	−3.04 (−6.88, 0.85)	−1.46 (−5.78, 2.87)	−0.58 (−1.66, 0.50)
Performance status ≥2	6.35 (0.28, 12.40)	5.88 (1.41, 10.37)*	3.63 (−1.39, 8.68)	5.94 (0.35, 11.54)	6.13 (−0.13, 12.41)	1.38 (−0.19, 2.95)
High-risk cytogenetics	−0.24 (−3.81, 3.35)	0.89 (−1.77, 3.55)	0.87 (−2.12, 3.86)	−1.92 (−5.25, 1.40)	−0.43 (−4.15, 3.31)	−0.16 (−1.09, 0.78)
Extramedullary disease	−3.79 (−8.55, 0.95)	−0.09 (−3.63, 3.44)	−0.44 (−4.43, 3.54)	−2.83 (−7.27, 1.59)	−3.24 (−8.23, 1.72)	−1.05 (−2.29, 0.19)
High marrow burden	−1.54 (−5.66, 2.58)	−1.59 (−4.64, 1.48)	−2.41 (−5.83, 1.05)	−0.47 (−4.30, 3.36)	−2.98 (−7.27, 1.32)	−0.64 (−1.72, 0.43)
Prior anti-BCMA therapy	1.88 (−4.62, 8.35)	5.55 (0.72, 10.38)*	4.71 (−0.72, 10.14)	−1.84 (−7.87, 4.21)	2.60 (−4.18, 9.35)	0.27 (−1.42, 1.96)
Pre-D7	0.18 (0.09, 0.26)***	−0.03 (−0.09, 0.03)	−0.04 (−0.12, 0.03)	0.05 (−0.02, 0.13)	<0.01 (−0.09, 0.09)	<0.01 (−0.02, 0.02)
Post-D7	−0.10 (−0.12, −0.07)***	−0.02 (−0.04, <0.01)	−0.04 (−0.07, −0.02)***	−0.03 (−0.05, −0.01)*	−0.05 (−0.07, −0.02)**	**−0.01 (−0.02**, < 0.01)**
Treatment	−2.84 (−6.83, 1.19)	0.27 (−2.68, 3.27)	−1.49 (−4.82, 1.90)	2.33 (−1.36, 6.06)	−2.97 (−7.13, 1.23)	−0.72 (−1.76, 0.32)
Pre-D7 × Treatment	−0.06 (−0.19, 0.06)	−0.01 (−0.10, 0.08)	0.02 (−0.08, 0.13)	−0.07 (−0.17, 0.04)	−0.08 (−0.20, 0.06)	−0.01 (−0.04, 0.02)
Post-D7 × Treatment	0.02 (−0.02, 0.05)	<0.01 (−0.03, 0.03)	0.01 (−0.03, 0.04)	0.01 (−0.02, 0.04)	0.02 (−0.02, 0.07)	<0.01 (−0.01, 0.01)

Reference groups for categorical variables are as follows: non-Hispanic White race and ethnicity, not married, performance status <2, no high-risk cytogenetics, no extramedullary disease, no high bone marrow burden, and no prior anti-BCMA therapy. Reference group for treatment was cilta-cel. *n* = 8 participants were excluded from the models due to missing covariate data (*n* = 6 missing high bone marrow burden; *n* = 5 missing high-risk cytogenetics; *n* = 1 missing extramedullary disease, not mutually exclusive) or other race/ethnicity group (*n* = 1).

Values reported are betas with 95% confidence intervals.

Significant values are indicated with bold text; *p < 0.05, **p < 0.01, ***p < 0.001.

**Table 3:** Results of piecewise growth curve models examining changes in PROMIS physical, social, and cognitive function scores by treatment group over time.

	Physical function	Social function	Cognitive function
(Intercept)	50.77 (36.8, 64.67)***	52.91 (38.14, 67.69)***	46.27 (35.83, 56.70)***
Age	−0.15 (−0.36, 0.06)	−0.12 (−0.34, 0.10)	0.06 (−0.10, 0.22)
Non-Hispanic Black	−4.58 (−9.4, 0.28)	−3.54 (−8.67, 1.64)	−0.71 (−4.37, 2.98)
Hispanic	−1.25 (−5.73, 3.21)	1.58 (−3.18, 6.34)	−1.66 (−5.01, 1.69)
Married	−0.35 (−4.39, 3.67)	−1.31 (−5.59, 2.98)	1.36 (−1.72, 4.40)
Performance status ≥2	−5.65 (−11.5, 0.21)	−5.53 (−11.76, 0.72)	−4.06 (−8.48, 0.37)
High-risk cytogenetics	−0.55 (−4.03, 2.93)	0.17 (−3.55, 3.86)	−0.18 (−2.81, 2.44)
Extramedullary disease	2.45 (−2.21, 7.15)	2.28 (−2.66, 7.23)	2.35 (−1.13, 5.84)
High marrow burden	1.54 (−2.47, 5.54)	2.07 (−2.19, 6.34)	1.21 (−1.80, 4.23)
Prior anti-BCMA therapy	−2.06 (−8.37, 4.24)	−1.61 (−8.33, 5.12)	−1.75 (−6.51, 3.00)
Pre-D7	−0.15 (−0.23, −0.08)***	−0.15 (−0.23, −0.07)***	−0.02 (−0.10, 0.06)
Post-D7	0.06 (0.03, 0.08)***	0.05 (0.03, 0.07)***	0.04 (0.02, 0.07)**
Treatment	1.35 (−2.55, 5.22)	2.69 (−1.45, 6.80)	0.8 (−2.19, 3.78)
Pre-D7 x Treatment	−0.03 (−0.14, 0.08)	0.03 (−0.08, 0.14)	0.04 (−0.08, 0.16)
Post-D7 x Treatment	0.02 (−0.02, 0.05)	0.01 (−0.02, 0.05)	−0.05 (−0.09, −0.02)**

Reference groups for categorical variables are as follows: non-Hispanic White race and ethnicity, not married, performance status <2, no high-risk cytogenetics, no extramedullary disease, no high bone marrow burden, and no prior anti-BCMA therapy. Reference group for treatment was cilta-cel. *n* = 8 participants were excluded from the models due to missing covariate data (*n* = 6 missing high bone marrow burden; *n* = 5 missing high-risk cytogenetics; *n* = 1 missing extramedullary disease, not mutually exclusive) or other race/ethnicity group (*n* = 1).

Values reported are betas with 95% confidence intervals.

Significant values are indicated with bold text; *p < 0.05, **p < 0.01, ***p < 0.001.

Trajectories were significantly different between groups for social well-being pre-D7 (β = 0.05, *p* = 0.046) and cognitive function post-D7 (β = −0.05, *p* = 0.004). Specifically, social well-being worsened pre-D7 among cilta-cel recipients (β = −0.04, *p* = 0.01) but did not change pre-D7 for ide-cel (β = 0.01, *p* = 0.723) and did not change post-D7 for either group (Fig. 1c). Cognitive function did not change for either group pre-D7 but significantly improved post-D7 for cilta-cel (β = 0.04, *p* = 0.001) and did not change post-D7 for ide-cel (β = −0.01, *p* = 0.379) (Fig. 3c).

In both treatment groups, many PROs significantly worsened pre-D7 and then significantly improved post-D7: overall HRQOL (Fig. 1a), physical well-being (Fig. 1b), functional well-being (Fig. 1e), fatigue (Fig. 2a), physical function (Fig. 3a), and social function (Fig. 3b). Several PROs did not change pre-D7 and then significantly improved post-D7: anxiety (Fig. 2c), sleep disturbance (Fig. 2d), pain interference (Fig. 2e), and pain intensity (Fig. 2f). Emotional well-being improved pre-D7 and continued to improve post-D7 (slope change *p* = 0.10) (Fig. 1d). Depression did not change pre- or post-D7 (Fig. 2b).

In total, 8 participants were excluded from the piecewise growth curve models due to missing covariate data (*n* = 7) or identifying as a race or ethnicity other than non-Hispanic White, non-Hispanic Black, or Hispanic (*n* = 1). Excluded and included participants did not significantly differ on any baseline characteristics or PROs.

### Changes in PROs from baseline to D90

Supplementary Fig. 2 shows a forest plot of effect sizes representing changes in average PRO scores from baseline to D90 across groups. Several PROs significantly improved from baseline to D90, including overall HRQOL (Cohen’s d = 0.30 small-to-medium), physical well-being (Cohen’s d = 0.37 small-to-medium), emotional well-being (Cohen’s d = 0.50 medium), depression (Cohen’s d = −0.23 small), anxiety (Cohen’s d = −0.32 small-to-medium), pain interference (Cohen’s d = −0.44 small-to-medium), and pain intensity (Cohen’s d = −0.43 small-to-medium). Change in fatigue from baseline to D90 indicated potential improvement (Cohen’s d = −0.21, small). No other average PRO scores were significantly different from baseline to D90, indicating maintenance or return to baseline values.

## Discussion

This study was the first to compare PRO trajectories among patients with RRMM receiving standard of care ide-cel and cilta-cel CAR-T with serial assessments starting pre-treatment and continuing through 90 days post-treatment. For most PROs, ide-cel and cilta-cel showed similar trajectories, with PROs initially worsening or stable pre-D7 and then improving post-D7. Differences between ide-cel and cilta-cel emerged for social well-being and cognitive function.

Most PROs initially worsened or were stable during the acute CAR-T treatment period (pre-D7) and significantly improved to baseline levels or better during the post-treatment recovery period (post-D7), with no differences by treatment. Emotional well-being showed the largest improvement from baseline to D90 (medium effect), with smaller improvements noted for overall HRQOL, physical well-being, anxiety, pain interference, physical function, and depression. The KarMMa and CARTITUDE-1 clinical trials of ide-cel and cilta-cel found similar PRO benefits in the first three months post-treatment for important PRO domains including overall HRQOL, fatigue, pain, and physical function [12, 14]. In qualitative interviews, CARTITUDE-1 participants described their HRQOL and symptom improvements as “extremely meaningful,” and the subsequent treatment-free period allowed them greater independence, better social function, and the opportunity to return to work [37]. In prior studies, our team showed that CAR-T safety and clinical outcomes in standard of care are similar to those in clinical trials [38, 39]. The current findings expand this work and indicate that PRO benefits in the first 90 days after standard of care CAR-T are also similar to those reported in clinical trials. Moreover, comparable PRO trajectories between treatments are encouraging, as patients receiving either FDA-approved CAR-T may expect similar PRO benefits. This is notable, because our team previously showed that cilta-cel is associated with better treatment outcomes (i.e., treatment responses, progression-free and overall survival) but higher incidence of certain toxicities in standard of care [20]. Differential clinical and safety outcomes put physicians in the challenging position of balancing treatment efficacy with risk of toxicities based on individual patient characteristics when making CAR-T treatment decisions. These differences do not appear to extend to most PROs. Patients may be comforted by the knowledge that they can expect similar PRO benefits on average within the first 90 days post-treatment regardless of which CAR-T they receive.

One of the few differences in PRO trajectories that emerged was related to social well-being; pre-D7 social well-being worsened among cilta-cel recipients but not ide-cel, and post-D7 social well-being did not change for either group. In our previous study of ide-cel in standard of care, there were no changes in social well-being through 90 days post-CAR-T [19]. A recent meta-analysis of the effects of CAR-T for multiple myeloma, lymphoma, and leukemia found that, across diagnoses and CAR-T regimens, social function did not change within three months but improved at longer-term follow-up (i.e., more than six months post-treatment) [40]. Factors such as caregiver dynamics and baseline social support could affect social well-being, but were not assessed in this study. Future studies should further investigate the potential difference in the effects of CAR-T on social well-being and explore these and other potential mechanisms.

This study also found group differences in the trajectories of cognitive function; pre-D7 cognitive function did not change for either group, and post-D7 cognitive function significantly improved for cilta-cel recipients but not ide-cel. Several prior studies have assessed cognitive function among CAR-T recipients using objective and subjective methods due to the risk of neurotoxicity. Among patients with large B-cell lymphoma, self-reported cognitive function did not change within three months of CAR-T [41, 42]. Similar to social function, a recent meta-analysis concluded that self-reported cognitive function did not change within three months of CAR-T for multiple myeloma, lymphoma, and leukemia, but later improved (i.e., more than six months post-treatment) [40]. In general, RRMM patients receiving cilta-cel have higher risk of delayed neurotoxicity and non-ICANS neurotoxicity relative to ide-cel [20]. Indeed, in our sample, cilta-cel recipients (vs. ide-cel) had higher incidence of non-ICANS neurotoxicity (14% vs. 0%). Thus, the possibility of improved cognitive function with cilta-cel is intriguing. Treatment-related inflammation is often implicated when assessing cognitive impairment in cancer and is particularly relevant for CAR-T, given the “inflammatory storm” observed post-treatment [43]. Future studies examining how CAR-T affects cognitive function should consider the role of inflammation and how it changes throughout treatment as a potential explanatory mechanism. Examining sub-types of cognitive function (e.g., processing speed, memory) and how they are affected could offer additional insights into potential mechanisms.

### Strengths and limitations

This was a prospective study with serial collection of PROs using psychometrically robust measures. The inclusion of equal proportions of ide-cel and cilta-cel recipients allowed for the first comparison of PROs between FDA-approved CAR-T treatments. Similar to our prior work [19], we had excellent patient engagement, with >90% retention at D90. This offers additional strong support for the feasibility of collecting PRO data from this unique population in real-world settings.

There were several limitations. Recruitment was limited to a single institution, and participants were mostly non-Hispanic White and highly educated. These characteristics are reflective of the patients who had access to and received CAR-T at an NCI-designated comprehensive cancer center. Racial and ethnic minority patients are underrepresented in RRMM CAR-T clinical trials and are less likely to receive CAR-T in standard of care [44, 45]. Future studies should replicate our findings among more diverse cohorts that are representative of the broader RRMM population. Participants were not randomized to a treatment group, and group comparisons may be limited by potential selection bias and confounding. In addition, analyses were limited to PROs collected through 90 days post-CAR-T. PRO trajectories by treatment may diverge with longer follow-up in the context of delayed toxicities beyond D90 and differential clinical outcomes (e.g., progression-free survival) [20]. This should be investigated in future studies with longer follow-up, with close attention to the occurrence of late onset toxicities.

## Conclusions

This was the first study to examine longitudinal PROs after ide-cel vs. cilta-cel CAR-T among RRMM patients in standard of care. Patients receiving ide-cel and cilta-cel had comparable PRO trajectories, with most PROs worsening or stable through D7 and then significantly improving to baseline levels or better by D90. This was similar to observations from RRMM CAR-T clinical trials, supporting the conclusion that patients treated in standard of care may expect similar PRO benefits as reported in clinical trials regardless of the CAR-T treatment they receive. Potential differences by treatment for social well-being and cognitive function should be investigated further, as should potential differences in PRO trajectories with longer-term follow-up.

## Supplementary information

Supplemental Materials

## Data Availability

The data that support the findings of this study are available from the corresponding author upon reasonable request.

## Code availability

Code for these analyses is stored in a private Moffitt GitHub repository (https://github.com/Moffitt-Cancer-Center/CARTMM-PRO-By-Treatment-BMT-Oswald) and available from the corresponding author upon reasonable request.
