# Supplementary material for: Patient-reported outcomes after idecabtagene vicleucel vs. ciltacabtagene autoleucel CAR-T for multiple myeloma
Source: Bone Marrow Transplant. 2026 May 11;61(7):864–72. doi: 10.1038/s41409-026-02899-w (PMC13349860; doi:10.1038/s41409-026-02899-w)

**Supplemental Table 1.** Baseline demographic and clinical characteristics for patients who were eligible and approached but declined to participate vs. patients who enrolled in the study.

|  | **Declined (n=62)** | **Enrolled (n=99)** | **p-value*** |
| --- | --- | --- | --- |
| Age, M (SD) | 68.24 (9.90) | 67.17 (9.62) | 0.46 |
| Median, range | 70, 41-88 | 66, 46-84 |  |
| Sex, n (%) |  |  | 0.26 |
| Female | 30 (48) | 39 (39) |  |
| Male | 32 (52) | 60 (61) |  |
| Race/ethnicity, n (%) |  |  | 0.70 |
| Non-Hispanic White | 44 (73) | 67 (68) |  |
| Non-Hispanic Black | 6 (10) | 16 (16) |  |
| Hispanic | 9 (15) | 15 (15) |  |
| Other | 1 (2) | 1 (1) |  |
| High marrow burden, n (%) | 15 (38) | 22 (24) | 0.10 |
| Missing | 22 | 6 |  |
| Extramedullary disease, n (%) | 11 (18) | 15 (15) | 0.68 |
| Missing | 0 | 1 |  |
| ECOG performance status ≥2, n (%) | 14 (23) | 9 (9) | 0.01 |
| High-risk cytogenetic abnormalities,^†^ n (%) |  |  |  |
| Any | 24 (60) | 35 (37) | 0.02 |
| del(17p) | 20 (50) | 22 (23) | 0.002 |
| t(4;14) | 9 (23) | 8 (9) | 0.03 |
| t(14;16) | 0 (0) | 8 (9) | 0.11 |
| Missing | 22 | 5 |  |
| Bridging therapy, n (%) | 47 (76) | 76 (77) | 0.89 |
| Response to bridging therapy,^‡^ n (%) |  |  | 0.53 |
| PR or better | 3 (6) | 10 (13) |  |
| SD/PD | 41 (87) | 61 (80) |  |
| Unknown response | 3 (6) | 5 (7) |  |
| Prior therapies, M (SD) | 5.15 (1.66) | 5.12 (1.51) | 0.89 |
| Median, range | 5, 3-13 | 5, 3-10 |  |
| Prior anti-BCMA treatment, n (%) | 2 (3) | 7 (7) | 0.48 |
| Penta-refractory, n (%) | 15 (24) | 14 (14) | 0.11 |

Note: BCMA, B-cell maturation antigen; ECOG, Eastern Cooperative Oncology Group, PD, progressive disease; PR, partial response; SD, stable disease. High marrow burden was defined as ≥50% CD138-positive plasma cells in pre-treatment bone marrow core biopsy. High-risk cytogenetics were defined as the presence of del(17p), t(4;14), and/or t(14;16) at any time before CAR-T infusion. Penta-refractory was defined as refractory to immunomodulatory agents, proteasome inhibitors or anti-CD38 antibodies. Percentages may not sum to 100 due to rounding. *Wilcoxon rank sum tests for age, and prior therapies, Fisher's exact tests for categorical variables with cell counts <5, and chi-square tests for all other categorical variables. ^†^Categories are not mutually exclusive. ^‡^Only reported for individuals who received bridging therapy.

**Supplemental Table 2.** Baseline participant demographic and clinical characteristics overall and by CAR-T treatment group.

|  | **Overall (N=99)** | **Ide-cel (n=49)** | **Cilta-cel (n=50)** | **p-value*** |
| --- | --- | --- | --- | --- |
| Age, M (SD) | 67.17 (9.62) | 71.17 (9.93) | 63.24 (7.53) | <0.001 |
| Median, range | 66, 46-84 | 73, 46-84 | 64, 46-80 |  |
| Sex, n (%) |  |  |  | 0.94 |
| Female | 39 (39) | 20 (41) | 19 (38) |  |
| Male | 60 (61) | 29 (59) | 31 (62) |  |
| Race/ethnicity, n (%) |  |  |  | 0.07 |
| Non-Hispanic White | 67 (68) | 33 (69) | 34 (68) |  |
| Non-Hispanic Black | 16 (16) | 11 (23) | 5 (10) |  |
| Hispanic | 15 (15) | 4 (8) | 11 (22) |  |
| Other | 1 | 1 | 0 |  |
| Marital status, n (%) |  |  |  | 0.07 |
| Married | 79 (80) | 35 (71) | 44 (88) |  |
| Not married | 20 (20) | 14 (29) | 6 (12) |  |
| Educational status, n (%) |  |  |  | 0.19 |
| College graduate | 53 (54) | 30 (61) | 23 (46) |  |
| Not college graduate | 46 (47) | 19 (39) | 27 (54) |  |
| Household Income, n (%) |  |  |  | 0.80 |
| <$40,000 | 21 (26) | 11 (28) | 10 (23) |  |
| ≥$40,000 | 61 (74) | 28 (72) | 33 (77) |  |
| Missing | 17 | 10 | 7 |  |
| CCI, M (SD) | 1.33 (1.55) | 1.57 (1.75) | 1.09 (1.28) | 0.25 |
| Median, range | 1, 0-7 | 1, 0-7 | 1, 0-6 |  |
| Missing | 6 | 2 | 4 |  |
| High marrow burden, n (%) | 22 (24) | 8 (18) | 14 (29) | 0.35 |
| Missing | 6 | 5 | 1 |  |
| Extramedullary disease, n (%) | 15 (15) | 7 (14) | 8 (16) | >0.99 |
| Missing | 1 | 0 | 1 |  |
| ECOG performance status ≥2, n (%) | 9 (9) | 5 (10) | 4 (8) | 0.74 |
| High-risk cytogenetic abnormalities,^†^ n (%) |  |  |  |  |
| Any | 35 (37) | 17 (39) | 18 (36) | 0.96 |
| del(17p) | 22 (23) | 13 (30) | 9 (18) | 0.28 |
| t(4;14) | 8 (9) | 3 (7) | 5 (10) | 0.72 |
| t(14;16) | 8 (9) | 3 (7) | 5 (10) | 0.72 |
| Missing | 5 | 5 | 0 |  |
| Bridging therapy, n (%) | 76 (77) | 38 (78) | 38 (76) | 0.86 |
| Response to bridging therapy,^‡^ n (%) |  |  |  | 0.33 |
| PR or better | 10 (13) | 3 (8) | 7 (18) |  |
| SD/PD | 61 (80) | 33 (87) | 28 (74) |  |
| Unknown response | 5 (7) | 2 (5) | 3 (8) |  |
| Prior therapies, M (SD) | 5.12 (1.51) | 5.31 (1.57) | 4.94 (1.45) | 0.20 |
| Median, range | 5, 3-10 | 5, 3-9 | 4, 3-10 |  |
| Prior anti-BCMA treatment, n (%) | 7 (7) | 5 (10) | 2 (4) | 0.27 |
| Penta-refractory, n (%) | 14 (14) | 7 (14) | 7 (14) | >0.99 |

Note: BCMA, B-cell maturation antigen; CCI, Charlson Comorbidity Index; ECOG, Eastern Cooperative Oncology Group, PD, progressive disease; PR, partial response; SD, stable disease. High marrow burden was defined as ≥50% CD138-positive plasma cells in pre-treatment bone marrow core biopsy. High-risk cytogenetics were defined as the presence of del(17p), t(4;14), and/or t(14;16) at any time before CAR-T infusion. Penta-refractory was defined as refractory to immunomodulatory agents, proteasome inhibitors or anti-CD38 antibodies. Percentages may not sum to 100 due to rounding. *Wilcoxon rank sum tests for age, prior therapies, and CCI, Fisher's exact tests for categorical variables with cell counts <5, and chi-square tests for all other categorical variables. ^†^Categories are not mutually exclusive. ^‡^Only reported for participants who received bridging therapy.

**Supplemental Table 3.** Safety and clinical outcomes in the first 90 days post-CAR-T infusion overall and by CAR-T treatment group.

|  | **Overall (N=99)** | **Ide-cel (n=49)** | **Cilta-cel (n=50)** | **p-value*** |
| --- | --- | --- | --- | --- |
| Hospitalization, days; M (SD) | 9.08 (5.98) | 10.73 (5.39) | 7.46 (6.14) | <0.001 |
| Median, range | 8, 0-34 | 9, 6-30 | 5, 0-34 |  |
| CRS, n (%) |  |  |  |  |
| Any grade | 91 (92) | 43 (88) | 48 (96) | 0.16 |
| Grade ≥2 | 19 (19) | 8 (16) | 11 (22) | 0.64 |
| ICANS, n (%) |  |  |  |  |
| Any grade | 19 (19) | 12 (25) | 7 (14) | 0.29 |
| Grade ≥2 | 11 (11) | 7 (14) | 4 (8) | 0.36 |
| Non-ICANS neurotoxicity, n (%) |  |  |  |  |
| Any | 7 (7) | 0 (-) | 7 (14) | 0.012 |
| Parkinsonism | 1 (1) | 0 (-) | 1 (2) | >0.99 |
| Any infection, n (%) | 48 (48) | 21 (43) | 27 (54) | 0.36 |
| Cytopenias,^†^ n (%) |  |  |  |  |
| Any | 96 (100) | 48 (100) | 48 (100) | - |
| Severe | 81 (85) | 43 (90) | 38 (81) | 0.36 |
| Best overall response by day 90, n (%) |  |  |  | 0.07 |
| PR or better | 87 (88) | 40 (82) | 47 (94) |  |
| SD/PD | 12 (12) | 9 (18) | 3 (6) |  |
| Best CR or better by day 90, n (%) |  |  |  | 0.08 |
| CR or better | 64 (65) | 27 (55) | 37 (74) |  |
| Less than CR | 35 (35) | 22 (45) | 13 (26) |  |

Note: CR, complete response; CRS, cytokine release syndrome; ICANS, immune effector cell-associated neurotoxicity syndrome; PD, progressive disease; PR, partial response; SD, stable disease. Percentages may not sum to 100 due to rounding. *Wilcoxon rank sum tests for hospitalization, Fisher's exact tests for categorical variables with cell counts <5, and chi-square tests for all other categorical variables. ^†^Cytopenias include those reported at D30 and D90.

**Supplemental Table 4.** Unadjusted FACT-G means (standard deviations) by treatment group at each timepoint.

| **Timepoint** | **Treatment** | **n** | **Overall**  **HRQOL** | **Physical**  **Well-being** | **Social**  **Well-being** | **Emotional**  **Well-being** | **Functional**  **Well-being** |
| --- | --- | --- | --- | --- | --- | --- | --- |
| BL | Ide-cel | 49 | 79.31 (14.24) | 20.26 (4.88) | 22.71 (4.73) | 19.03 (3.40) | 17.31 (5.60) |
| BL | Cilta-cel | 50 | 81.01 (15.26) | 20.82 (5.59) | 24.33 (3.09) | 18.52 (4.60) | 17.34 (6.03) |
| D0 | Ide-cel | 40 | 80.59 (13.81) | 19.77 (5.69) | 23.69 (4.26) | 19.97 (4.09) | 16.97 (5.75) |
| D0 | Cilta-cel | 47 | 81.88 (13.13) | 21.12 (5.12) | 23.89 (3.49) | 20.00 (3.75) | 16.87 (6.57) |
| D7 | Ide-cel | 45 | 73.79 (14.30) | 17.62 (5.96)^a^ | 22.80 (4.75) | 19.07 (3.92) | 14.30 (4.82) |
| D7 | Cilta-cel | 42 | 73.24 (16.32) | 16.71 (7.07)^a^ | 23.28 (4.05) | 19.26 (4.19) | 14.00 (6.67) |
| D14 | Ide-cel | 39 | 80.13 (15.11) | 21.22 (5.29) | 22.86 (4.80) | 20.32 (3.82) | 15.73 (6.09) |
| D14 | Cilta-cel | 45 | 78.40 (16.68) | 19.82 (5.78) | 23.05 (4.98) | 20.30 (3.66) | 15.23 (6.79) |
| D21 | Ide-cel | 42 | 80.81 (13.06) | 21.39 (4.53) | 23.24 (5.34) | 20.28 (3.04) | 15.90 (5.16) |
| D21 | Cilta-cel | 41 | 82.52 (14.66) | 22.10 (4.20) | 23.06 (4.48) | 21.12 (3.00) | 16.24 (7.44) |
| D30 | Ide-cel | 43 | 82.09 (12.33) | 21.34 (4.75) | 23.52 (4.79) | 20.98 (2.64) | 16.26 (4.71) |
| D30 | Cilta-cel | 44 | 82.35 (16.83) | 21.90 (4.96) | 23.51 (4.21) | 20.57 (4.02) | 16.37 (7.25) |
| D60 | Ide-cel | 40 | 85.32 (12.67) | 23.04 (3.74) | 23.22 (4.24) | 20.72 (3.16) | 18.34 (4.99) |
| D60 | Cilta-cel | 44 | 85.55 (12.80) | 22.70 (4.93) | 23.62 (3.54) | 21.14 (2.70) | 18.10 (6.09) |
| D90 | Ide-cel | 44 | 83.28 (14.98) | 22.21 (4.62) | 23.01 (4.69) | 20.76 (3.14) | 17.29 (6.07) |
| D90 | Cilta-cel | 45 | 85.11 (14.06) | 22.49 (5.01) | 23.90 (3.37) | 20.72 (2.76) | 18.01 (6.50) |

Note. ^a^Average score is below the threshold indicating clinically low HRQOL (overall HRQOL ≤70, physical well-being ≤18, social well-being ≤19, emotional well-being ≤15, and functional well-being ≤14).

**Supplemental Table 5.** Unadjusted PROMIS means (standard deviations) by treatment group at each timepoint.

| **Timepoint** | **Treatment** | **n** | **Fatigue** | **Depression** | **Anxiety** | **Sleep**  **Disturbance** | **Pain Interference** | **Pain**  **Intensity** | **Physical**  **Function** | **Social Function** | **Cognitive Function** |
| --- | --- | --- | --- | --- | --- | --- | --- | --- | --- | --- | --- |
| BL | Ide-cel | 49 | 54.21 (9.89) | 48.97 (8.03) | 50.74 (8.06) | 51.17 (8.09) | 56.29 (8.80)^a^ | 3.53 (2.44)^a^ | 42.04 (7.30)^a^ | 47.24 (9.81) | 51.75 (7.67) |
| BL | Cilta-cel | 50 | 51.58 (9.70) | 46.80 (7.35) | 50.25 (9.00) | 47.71 (8.71) | 53.93 (10.97) | 3.02 (2.71)^a^ | 44.11 (9.74)^a^ | 48.35 (9.38) | 52.74 (7.52) |
| D0 | Ide-cel | 40 | 55.53 (8.52)^a^ | 46.75 (8.54) | 50.16 (9.19) | 51.10 (9.77) | 53.54 (10.27) | 3.08 (2.69)^a^ | 41.16 (8.29)^a^ | 47.64 (8.88) | 50.45 (7.77) |
| D0 | Cilta-cel | 47 | 53.66 (9.59) | 46.05 (7.26) | 49.98 (8.89) | 48.28 (8.09) | 54.26 (10.65) | 2.70 (2.64) | 41.59 (9.68)^a^ | 45.33 (9.64) | 52.28 (8.97) |
| D7 | Ide-cel | 45 | 57.47 (8.97)^a^ | 47.88 (8.65) | 51.12 (10.33) | 52.94 (9.34) | 54.46 (11.14) | 2.76 (2.78) | 36.27 (8.18)^a^ | 42.77 (8.62)^a^ | 50.73 (7.99) |
| D7 | Cilta-cel | 42 | 58.89 (11.89)^a^ | 46.78 (8.32) | 49.50 (10.24) | 50.92 (9.36) | 55.79 (11.11)^a^ | 3.40 (2.81)^a^ | 37.64 (9.77)^a^ | 43.55 (11.28)^a^ | 49.71 (9.25) |
| D14 | Ide-cel | 39 | 56.54 (9.92)^a^ | 48.58 (8.52) | 49.02 (8.97) | 49.44 (9.61) | 51.04 (10.16) | 2.32 (2.67) | 39.37 (8.96)^a^ | 44.88 (9.31)^a^ | 52.51 (7.67) |
| D14 | Cilta-cel | 45 | 56.56 (12.01)^a^ | 46.24 (7.83) | 49.53 (8.72) | 49.58 (10.12) | 52.51 (10.79) | 2.49 (2.90) | 40.84 (9.98)^a^ | 44.82 (12.27)^a^ | 50.68 (9.15) |
| D21 | Ide-cel | 42 | 54.51 (9.05) | 48.44 (8.19) | 48.80 (8.85) | 48.37 (8.83) | 50.76 (9.89) | 2.12 (2.32) | 38.88 (8.61)^a^ | 45.13 (8.15) | 51.87 (7.48) |
| D21 | Cilta-cel | 41 | 52.97 (10.80) | 44.51 (6.62) | 45.85 (7.26) | 45.94 (8.97) | 50.05 (10.09) | 1.88 (2.15) | 41.86 (9.87)^a^ | 45.92 (10.77) | 51.68 (9.08) |
| D30 | Ide-cel | 43 | 55.20 (8.63)^a^ | 45.55 (7.30) | 48.20 (7.97) | 49.35 (7.88) | 53.05 (8.86) | 2.44 (2.23) | 40.03 (9.07)^a^ | 45.63 (7.58) | 52.73 (7.46) |
| D30 | Cilta-cel | 44 | 54.20 (10.22) | 44.86 (6.55) | 46.82 (8.83) | 46.91 (8.91) | 51.40 (10.84) | 1.95 (2.40) | 41.00 (9.05)^a^ | 46.22 (10.74) | 52.23 (8.37) |
| D60 | Ide-cel | 40 | 49.77 (9.26) | 46.77 (7.14) | 47.06 (8.01) | 49.94 (7.59) | 50.84 (8.85) | 2.33 (2.16) | 43.83 (8.74)^a^ | 48.75 (8.20) | 51.50 (9.25) |
| D60 | Cilta-cel | 44 | 48.75 (10.79) | 44.33 (5.54) | 45.40 (6.57) | 47.89 (8.81) | 51.51 (10.83) | 2.20 (2.59) | 44.32 (10.43)^a^ | 48.21 (10.54) | 53.95 (6.65) |
| D90 | Ide-cel | 44 | 52.00 (10.40) | 47.04 (7.65) | 48.06 (8.48) | 49.66 (9.26) | 52.44 (8.67) | 2.45 (2.37) | 42.54 (8.07)^a^ | 48.39 (8.36) | 50.76 (8.19) |
| D90 | Cilta-cel | 45 | 50.03 (10.63) | 45.21 (6.63) | 46.31 (7.25) | 46.31 (10.02) | 51.41 (11.46) | 2.13 (2.69) | 44.39 (10.99)^a^ | 48.12 (11.41) | 53.96 (7.54) |

Note. ^a^Average score is clinically meaningful (fatigue, depression, anxiety, sleep disturbance, and pain interference: 55-59 mild, 60-69 moderate, ≥70 severe; pain intensity: 1-2 mild, 3-5 moderate, ≥6 severe; physical, social, and cognitive function: ≤30 severe, 31-40 moderate, 41-45 mild).

**Supplemental Figure 1.** Participants’ flow through the study. Participants who were deceased before each timepoint were excluded from the denominator when calculating percentages.

**Supplemental Figure 2.** Cohen’s d effect sizes for unadjusted changes in PROs from baseline to D90 across CAR-T treatment groups. Effect sizes were interpreted as: 0.2 small; 0.3-0.5 small-to-medium; 0.5-0.8 medium; >0.8 large).


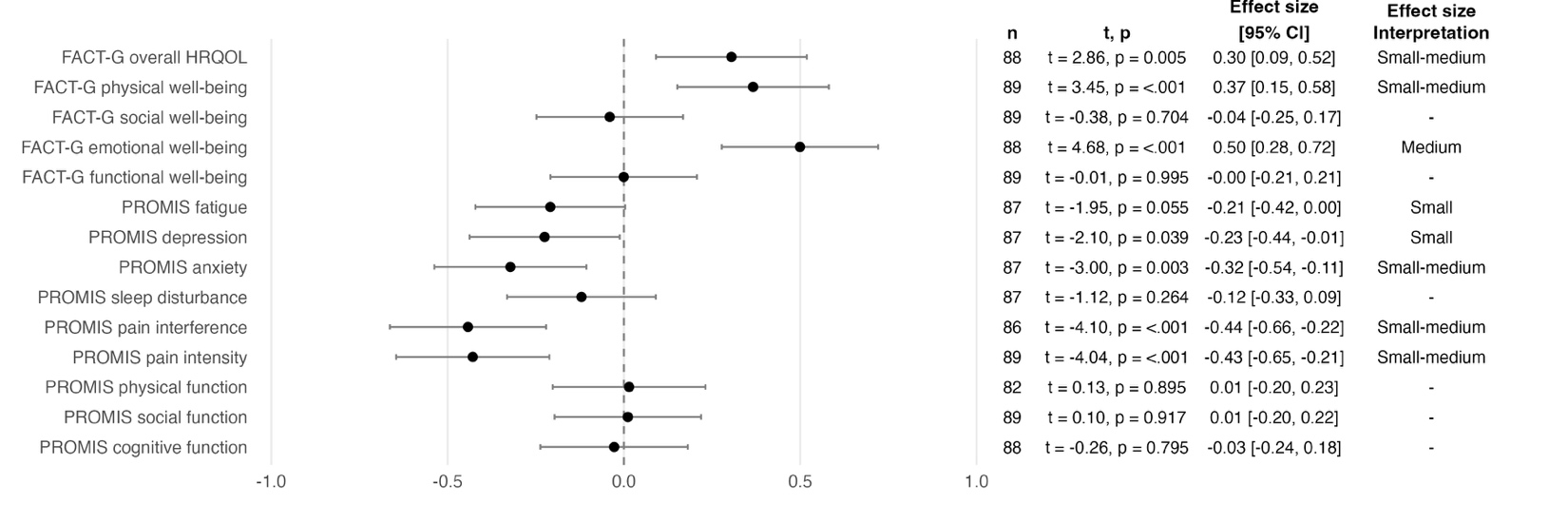

Supplement: Supplementary file 1 — Supplemental Materials [file 41409_2026_2899_MOESM1_ESM.docx]
